# Supplementary material for: The characteristics of spatial expansion and driving forces of land urbanization in counties in central China: A case study of Feixi county in Hefei city
Source: PLoS One. 2021 May 26;16(5):e0252331. doi: 10.1371/journal.pone.0252331 (PMC8153434; doi:10.1371/journal.pone.0252331)
Supplement: S1 Data — (DOCX) [file pone.0252331.s001.docx]

Changing Scale of urban land areas in Feixi 2002-2016

Changes in urban land in towns of Feixi county from 2002 to 2016

| Township | 2002 | | 2006 | | | 2009 | | | 2012 | | | 2016 | | | 2002-2016 |
| --- | --- | --- | --- | --- | --- | --- | --- | --- | --- | --- | --- | --- | --- | --- | --- |
|  | Urban land（hm^2^） | Proportion （%） | Urban land （hm^2^） | Proportion （%） | Average annual growth rate（%） | Urban land （hm^2^） | Proportion （%） | Average annual growth rate（%） | Urban land（hm^2^） | Proportion （%） | Average annual growth rate（%） | Urban land （hm^2^） | Proportion （%） | Average annual growth rate（%） | Average annual growth rate（%） |
| Taohua | 550.62 | 28.41 | 1085.1 | 28.98 | 18.48 | 2443 | 36.38 | 31.06 | 2920.7 | 36.13 | 6.13 | 3172.03 | 33.96 | 2.09 | 13.32 |
| Shangpai | 680.74 | 35.12 | 1065.26 | 28.45 | 11.85 | 1531.14 | 22.8 | 12.85 | 2160.07 | 26.72 | 12.15 | 2712.16 | 29.04 | 5.86 | 10.38 |
| Zipeng | 104.04 | 5.37 | 468.47 | 12.51 | 45.67 | 1040.76 | 15.5 | 30.48 | 1148.72 | 14.21 | 3.34 | 1521.65 | 16.29 | 7.28 | 21.12 |
| Huanggang | 111.44 | 5.75 | 219.29 | 5.86 | 18.44 | 474.08 | 7.06 | 29.3 | 523.61 | 6.48 | 3.37 | 576.92 | 6.18 | 2.45 | 12.46 |
| Sanhe | 132.99 | 6.86 | 238.64 | 6.37 | 15.74 | 299.58 | 4.46 | 7.87 | 324.6 | 4.02 | 2.71 | 358.97 | 3.84 | 2.55 | 7.35 |
| Guanting | 106.69 | 5.5 | 208.39 | 5.57 | 18.22 | 317.64 | 4.73 | 15.09 | 344.05 | 4.26 | 2.7 | 349.75 | 3.74 | 0.41 | 8.85 |
| Shannan | 141.61 | 7.31 | 208.4 | 5.57 | 10.14 | 247.1 | 3.68 | 5.84 | 264.64 | 3.27 | 2.31 | 237.27 | 2.54 | -2.69 | 3.76 |
| Fengle | 30.41 | 1.57 | 68.93 | 1.84 | 22.7 | 143.61 | 2.14 | 27.72 | 144.19 | 1.78 | 0.13 | 139.53 | 1.49 | -0.82 | 11.5 |
| Yandian | 17.33 | 0.89 | 54.44 | 1.45 | 33.13 | 63.54 | 0.95 | 5.28 | 81.95 | 1.01 | 8.85 | 110.86 | 1.19 | 7.84 | 14.17 |
| Mingchuan | 23.53 | 1.21 | 60.2 | 1.61 | 26.48 | 84.98 | 1.27 | 12.18 | 83.48 | 1.03 | -0.59 | 87.82 | 0.94 | 1.27 | 9.86 |
| Gaodian | 15.12 | 0.78 | 25.99 | 0.69 | 14.5 | 28.43 | 0.42 | 3.04 | 44.96 | 0.56 | 16.5 | 39.28 | 0.42 | -3.32 | 7.06 |
| Shishugang | 23.53 | 1.21 | 40.92 | 1.09 | 14.84 | 42.06 | 0.63 | 0.92 | 42.83 | 0.53 | 0.61 | 33.47 | 0.36 | -5.98 | 2.55 |

Land use change survey data of Feixi county Bureau of Land and Resources (2006-2016)：


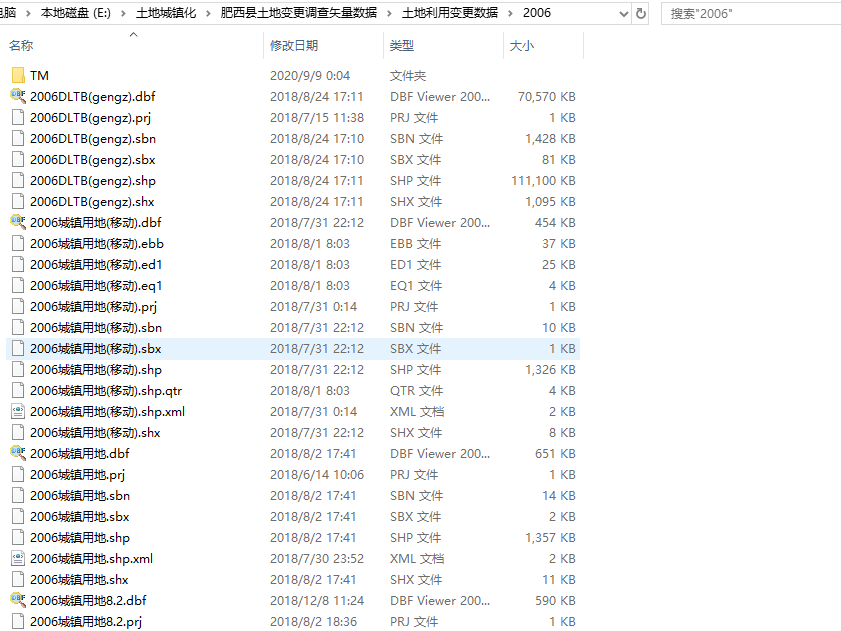


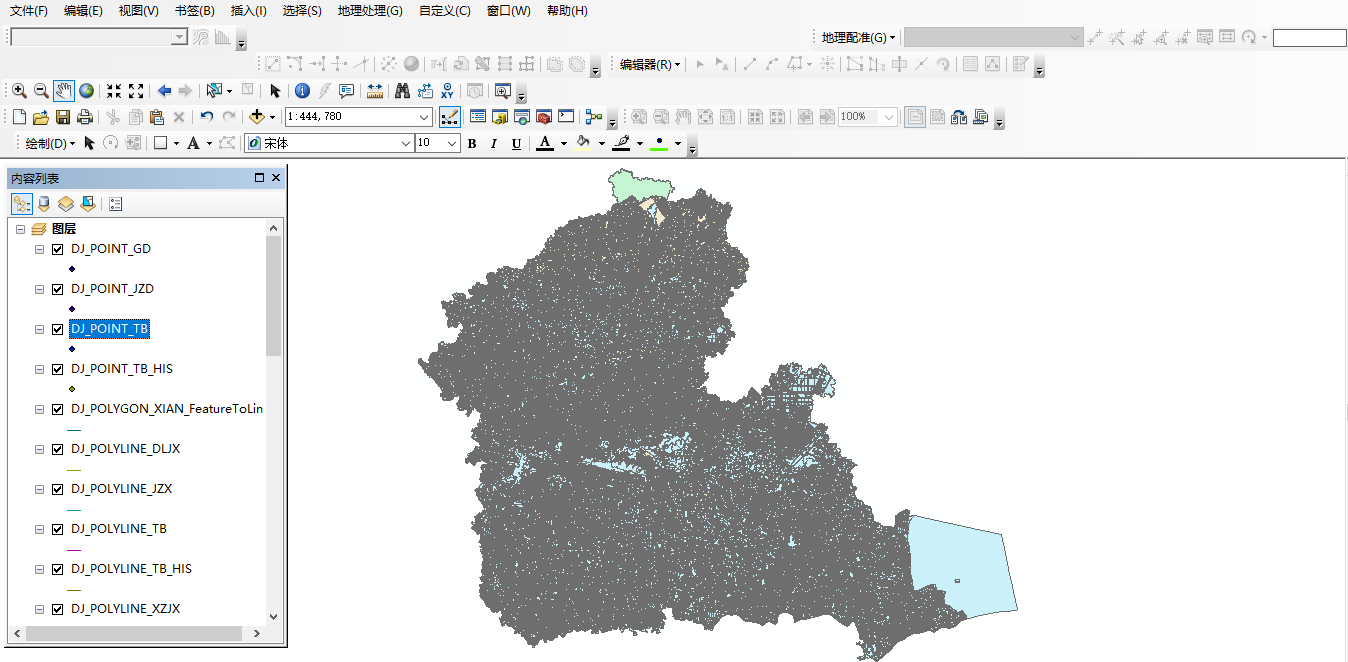


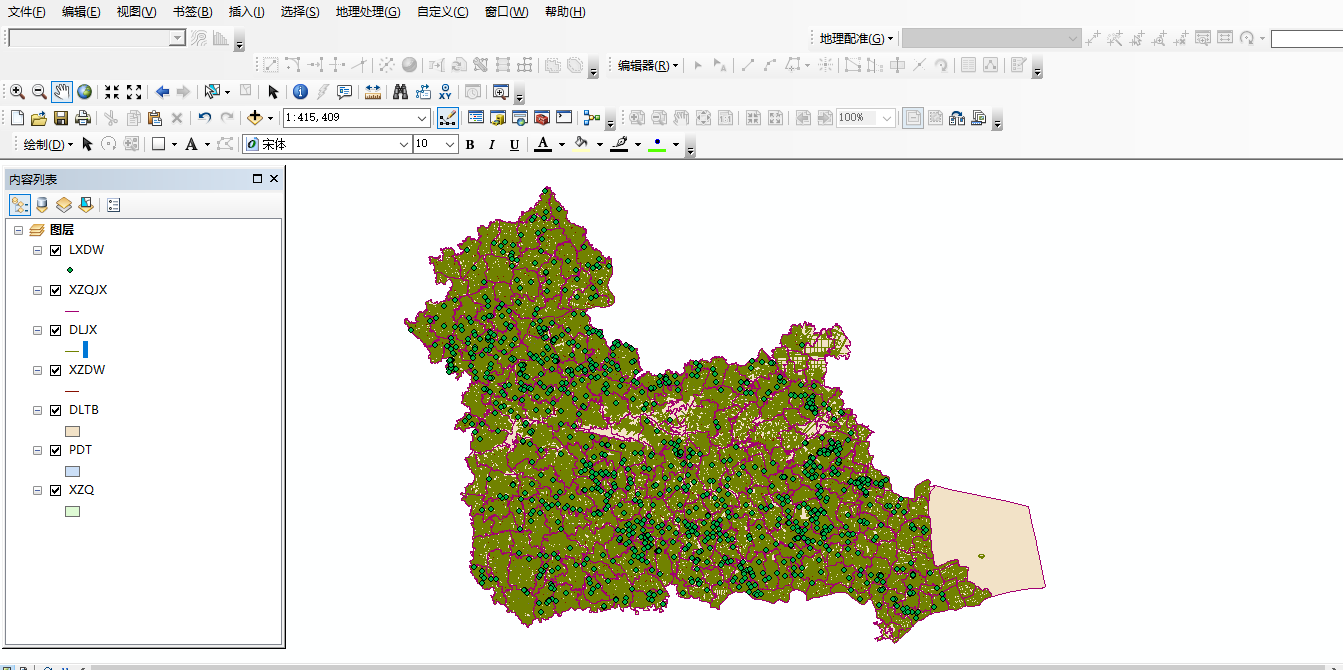


**Statistical Yearbooks of Feixi and Hefei：**

https://kns.cnki.net/kns/brief/result.aspx?dbprefix=CYFD
